# To Seek or Not to Seek Advice: Talking About Romantic Issues During Emerging Adulthood

**DOI:** 10.5964/ejop.v14i1.1454

**Published:** 2018-03-12

**Authors:** Semira Tagliabue, Maria Giulia Olivari, Cristina Giuliani, Emanuela Confalonieri

**Affiliations:** a Department of Psychology, Università Cattolica del Sacro Cuore, Brescia, Italy; b Department of Psychology, CRIdee, Università Cattolica del Sacro Cuore, Milano, Italy; c Department of Psychology, Università Cattolica del Sacro Cuore, Milano, Italy; Department of Psychology, Webster University Geneva, Geneva, Switzerland; University of Neuchâtel, Neuchâtel, Switzerland

**Keywords:** seeking advice, romantic relationships, parents, close friends, emerging adulthood, sexuality

## Abstract

The aim of the study was to explore whether and how emerging adults talk about their romantic relationships with their close others, especially their parents and friends, also considering gender differences. Data were collected via eight single-sex focus groups conducted with 50 Italian emerging adults (aged 18–25), and were analyzed using thematic analysis. Two main themes emerged. The first was labeled “to seek advice”, which was divided into three subthemes: “I look for different points of view,” “I treasure other people’s words,” and “I listen and then do it my own way.” The second theme was “to not seek advice,” which was divided into two subthemes: “I do not need comparison” and “I need to choose on my own.” The findings revealed that close friends, more than parents, are important interlocutors for discussions on romantic relationships, and few gender differences were found. Furthermore, we can speculate that emerging adults’ reasons for seeking advice or not could relate to their autonomy and relatedness needs.

Romantic relationships are a central issue in one’s transition to adulthood (Fincham & Cui, 2011), and the presence of a stable romantic relationship is linked with plans about leaving the parental home and marriage (Lanz & Tagliabue, 2007; Shulman & Connolly, 2013). However, in this period, romantic relationships are often unstable and fluid, characterized by break-ups and reconciliations, so that emerging adults experience difficulties in taking long-term decisions and commitment in the relationship itself (Dailey, Hampel, & Roberts, 2010). Communication and self-disclosure about romantic relationships have been found to be typical in adolescence, especially with friends and less with parents (Fry et al., 2014; Tishby et al., 2001), but very little research has been conducted on this topic during the transition to adulthood. Research on emerging adults seeking advice revealed that they look for parental support when they experience difficulties in different areas of their lives, but less with regard to how to manage their romantic relationships (Carlson, 2014). It could be that they prefer to turn to their friends to have support and to self-disclose about their romantic issues. Research also revealed gender differences in talking about romantic issues, with females communicating more frequently than males (Lefkowitz, Boone, & Shearer, 2004).

Few studies have been conducted on whether and how emerging adults talk about their romantic relationships with their parents and friends, especially in Italy. In order to fill this gap, the present qualitative study aimed to explore whether and how emerging adults talk about their romantic relationships with their parents and friends, also considering possible gender differences that have been found in adolescence.

## Romantic Relationships in Emerging Adulthood: Talking With Parents and Friends

According to Erikson (1950, 1963), the transition to adulthood is characterized by the dialectic of intimacy versus isolation: “Intimacy is the capacity to commit (one) self to concrete affiliations and partnerships and to develop the ethical strength to abide by such commitments, even though they may call for significant sacrifices and compromises” (Erikson, 1963, p. 263). Research confirms that intimacy is higher in emerging adulthood than in adolescence (Montgomery, 2005), so that developing a satisfactory relationship with a romantic partner constitutes an important developmental task of this period (Roisman, Masten, Coatsworth, & Tellegen, 2004; Schulenberg, Bryant, & O’Malley, 2004) and it could have long-term consequences regarding future choices (e.g., cohabitation, marriage, family planning, and parenthood) (Lanz & Tagliabue, 2007).

Furman and Winkles (2012) underlined that there is a developmental change in romantic relationships from adolescence to emerging adulthood, although these changes are fluid. The number of individuals involved in a romantic relationship increases during the transition to adulthood and the duration of these relationships also raises (Giordano, Flanigan, Manning, & Longmore, 2009; Seiffge-Krenke, 2003). Romantic relationships become the context where emerging adults develop their intimacy processes and attitudes towards lifelong commitment (Collins & van Dulmen, 2006; Seiffge-Krenke, 2003). Transitional choices such as cohabiting with a partner, being involved in a stable relationship, getting married, and choosing to have a baby represent central aspects in the transition to adulthood (Seiffge-Krenke, Luyckx, & Salmela-Aro, 2014). Moreover, sexual behaviors also increase during the transition to adulthood (Mosher, Chandra, & Jones, 2005) and become more linked to positive outcomes (Welsh, Haugen, Widman, Darling, & Grello, 2005). Indeed, although sexuality is still studied as a risk behavior in emerging adulthood (Vasilenko, Lefkowitz, & Maggs, 2012), it is also investigated concerning experimentation, including different ways of being involved in casual relationships (i.e., “hookups,” “one-night stands,” “friends with benefits,” and “booty calls”) (Claxton & van Dulmen, 2013) and it has been found that the frequency of sexual intercourse is associated to a higher relational commitment (Welsh et al., 2005). Some gender differences have also been found in the level of intimacy during the transition to adulthood: females present higher intimacy than males (Montgomery, 2005).

Given the centrality and importance of romantic relationships during emerging adulthood, romantic issues should be an important topic of communication, seeking advice and self-disclosure for emerging adults. However, few studies investigate whether and how emerging adults talk about romantic relationships with parents and friends.

During emerging adulthood, parents are still a major source of support in order to face uncertainties or difficulties (Oliveira, Mendonça, Coimbra, & Fontaine, 2014; Swartz, Kim, Uno, Mortimer, & O’Brien, 2011). Indeed, emerging adults often directly solicit parental support and advice in managing daily issues (Carlson, 2014). However, the advice mainly concerns working life balance and academic issues, and less social and relational issues regarding friendships, family life, and romantic relationships. Moreover, among relational issues, emerging adults solicit less advice about romantic relationships as compared to the advice requested for issues related to family or friend relationships. Carlson (2014) concluded that parental advice about romantic relationships, especially sexuality issues, is not often solicited, and even when it is, emerging adults do not follow it.

Some studies investigated possible differences in the way adolescents and emerging adults talk with parents or with friends regarding their romantic relationships and sexuality. Findings revealed that late adolescents and emerging adults disclose more with friends than with parents (Christopher, 2001; DiIorio, Kelley, & Hockenberry-Eaton, 1999; Lefkowitz & Espinosa-Hernandez, 2007). Often, adolescents feel embarrassed speaking with parents about their romantic relationships; therefore, they turn to their peers (Fry et al., 2014; Tishby et al., 2001), especially when they are experiencing negative events. Indeed, seeking advice and support from others when experiencing negative relational events with a romantic partner is a common way to reduce uncertainty (Planalp, Rutherford, & Honeycutt, 1988). Focus-group research conducted with emerging adults confirmed that emerging adults seek advice and communication about negative relational events in their romantic relationships in order to receive social support and reduce uncertainty (Vallade, Dillow, & Myers, 2016).

Regarding communication about sex, adolescents feel more comfortable to discuss sex topics with mothers than with fathers (DiIorio et al., 1999; Feldman & Rosenthal, 2000). Communication with friends about sex is characterized by a variety of topics (dating, feelings, abstinence, fertility issues, contraception, etc.); moreover, topics discussed and frequency of communication is linked with being sexual active and having liberal sexual attitudes (Lefkowitz et al., 2004).

Gender differences have been found in the way people talk with parents and friends about romantic issues. For instance, adolescent females feel more comfortable than males in talking with parents (Lefkowitz, Boone, Sigman, & Au, 2002; Raffaelli, Bogenschneider, & Flood, 1998) and with friends (DiIorio et al., 1999) about sex-related topics, although males face fewer difficulties in talking with fathers about sex-related topics than females (DiIorio et al., 1999). Lefkowitz and colleagues (2004) found similar findings in emerging adults: females feel more comfortable to speak with best friends about sex-related topics than males. When the topic of the research is the conversation about intimacy, some qualitative studies revealed that adolescent males are open to talk about their romantic desires with their male and female friends (Tolman, Spencer, Harmon, Rosen-Reynoso, & Striepe, 2004; Way, 2004), but another study conducted on male emerging adults underlined their difficulties in managing intimate topics in their conversation with friends (Korobov & Thorne, 2006).

Most of the cited research focused on adolescence. However, romantic partner in emerging adults’ social network acquires a central position, and also parental and friend relationships are changing. Parent-child relationship becomes more horizontal and friendly, and although it continues to have a central role (Parks, 2007), it is also characterized by a decrease in its quality (Flynn, Felmlee, & Conger, 2017). Thus, more research is needed in order to understand whether the patterns of communication about romantic issues are similar or different during the transition to adulthood compared with adolescence. Moreover, although in adolescence gender differences have been found (Lefkowitz et al., 2004), few studies explore possible gender differences during emerging adulthood considering both romantic and sex-related topics.

Literature about romantic issues’ self-disclosure during adolescence provides some insights, however the transition to adulthood is a different developmental phase (Arnett, 2000; Furman & Winkles, 2012) with specific features. For instance, emerging adulthood is characterized by a focus on intimacy (Erikson, 1963; Furman & Winkles, 2012), but also on the individuation process, in which emerging adults achieve autonomy and independence from close others, especially parents, still conserving a sense of connection with them (Blos, 1979; Mattanah, Hancock, & Brand, 2004). Thus, the way in which emerging adults talk with parents and friends about their romantic issues could also be influenced by the developmental processes characterizing this transition.

Despite the importance of romantic relationships in emerging adulthood, little research, especially in Italy, has been conducted on emerging adults’ communication about romantic relationships with close others. Emerging adulthood in Italy presents some unique properties such as the delay in leaving parental home, or the importance of romantic relationships for this role transition (Carrà, Lanz, & Tagliabue, 2014; Crocetti & Tagliabue 2016; Lanz & Tagliabue, 2007, 2014; Regalia, Lanz, Tagliabue, & Manzi, 2011). Research conducted in Italy revealed that being involved in a romantic relationship is specifically linked with future plans (e.g., leaving the parental home and having a baby; Lanz & Tagliabue, 2007) and with the emerging adults’ perception of what adulthood means; in particular people involved in a romantic relationship, more than single ones, think that adulthood is related to interdependence (an adult is characterized by being committed to long-term love relationships and making life-long commitments to others), role transitions (people married, with at least one child, settled into long-term career and owing a house are defined as adult people), respect of norms (adults avoid becoming drunk and do not have more than one sexual partner), and family capacities (adults are capable of caring for their family and children; Crocetti & Tagliabue, 2016).

Support from one’s family, both emotional and material, has been found to be relevant for emerging adults’ adjustment and identity (Crocetti & Meeus, 2014), although some findings revealed that romantic relationships are more important and affect the perception of the parent-child relationship’s quality, whereas the opposite is not true (Regalia et al., 2011). Friends’ support has also been found to be significantly linked with Italian emerging adults’ self-image (Tagliabue, Lanz, & Pozzi, 2006) and identity (Crocetti & Meeus, 2014); however, less research has addressed friendship during emerging adulthood in Italy.

## Aim of Current Research

Consequently, we qualitatively explored whether and how emerging adults talk with their close others about their romantic relationships, especially their parents and friends, also considering possible gender differences. We decided to include both emerging adults who are currently involved in romantic relationships and those who have been involved, but are now single. The reason for that choice is linked to the fact that people who are single today but have been involved in a romantic relationship in the past, have been forced to manage a break up. The experience of breaking up is, indeed, quite common during emerging adulthood, and it could have been an important reason for communication and self-disclosure with close others (Norona, Olmstead, & Welsh, 2017). We also decided to use single-sex focus groups in order to explore gender differences. In focus group sessions, a small number of people is invited to discuss regarding specific topics, and group social interaction is employed to enhance feedback on beliefs, experiences, and reactions of respondents (Gibbs, 1997), and to facilitate their self-disclosure through mutual support (Krueger & Casey, 2000). Moreover, focus groups often facilitate conversation on sensitive or high-involvement topics that people are usually reluctant to talk about (Zeller, 1993). Indeed, focus groups have been used previously in the investigation of dating and sexuality among adolescents and emerging adult groups (Noel, Ogle, Maisto, & Jackson, 2016; Olivari, Confalonieri, & Ionio, 2011; Olivari, Cuccì & Confalonieri, 2017; Regmi, van Teijlingen, Simkhada, & Acharya, 2011).

Our main research questions are:

1. Do emerging adults communicate and self-disclose about their romantic relationships (regarding both intimacy and sex-related topics) with parents and friends?
2. How do they do it?
3. Are there gender differences?

## Methods

### Participants

During 2014-15, participants were recruited for focus group participation using volunteer sampling. They comprised 50 university students (25 men and 25 women) aged 18–25 (*M*_age_ = 22.88, *SD* = 1.61) recruited from urban areas in Northern Italy. To collect data, we organized 8 single-sex focus groups (women: one group of 8, two groups of 6, one group of 5; men: one group of 7 and three groups of 6). The groups comprised a mix of ages.

Fifty-two percent of participants reported being in a romantic relationship at the time of the study. The average length of these relationships was 38.15 months (*SD* = 30.42). In 92% of the cases, participants were sexually active. The mean age of first sexual intercourse was 17.63 years (*SD* = 1.98).

### Materials

The focus group schedule was developed by the researchers for a larger study which aimed to explore how emerging adults manage and live their romantic and sexual relationships. We focused our attention on four questions soliciting participants’ views on seeking advice and sharing information regarding such relationships: 1) “With whom do you talk about issues concerning the person you have feelings for?” 2) “Do your friends’ opinions about the person who you have feelings for matter?” 3) “Do your parents’ opinions regarding the person who you have feelings for matter?” 4) “How do you feel when you talk about sexuality? With whom do you speak about it?” We only analyzed the narratives stimulated by these questions.

After the focus-group section, a questionnaire was individually administered to participants in order to collect participants’ socio-demographic information (e.g., sex, age) and romantic and sexual experience (e.g., presence of a romantic relationship, length, and sexual activity).

### Procedure

All participants provided their written consent to participate and for the tape-recording of the focus-group session. The participants were free to withdraw at any time and they were not compensated for participation.

Focus groups were conducted in a university suite by researchers with experience in the conduction of focus group, and they lasted for approximately 45 minutes each. Participants were first briefed about the aim of the research. The maintenance of confidentiality was assured by researchers and participants were asked to maintain the confidentiality of others in the group as well. The four questions on the schedule were asked; however, participants were also allowed to discuss the topic area more widely. Researchers emphasized that all responses were welcomed and that no response was considered right or wrong. The sessions were tape-recorded and later transcribed verbatim in Italian. The recruitment of participants continued until theoretical saturation was achieved and no other topics emerged during the focus groups. Specifically, we relied on the general notion of data saturation “as the point in data collection and analysis when new information produces little or no change to the codebook” (Guest, Bunce, & Johnson, 2006, pp. 65).

### Data Analysis

The transcripts from the focus groups were analyzed using thematic analysis, which is a method for identifying, analyzing, and reporting patterns (i.e., themes) within the data (Braun & Clarke, 2006). The thematic analysis was completed in four steps. First, the researchers familiarized themselves with the text by transcribing the recordings, reading these transcripts thoroughly, highlighting keywords and phrases, and then noting initial ideas. Second, they began the coding process, which involved organizing the data into themes and subthemes. The three authors first reviewed the transcripts separately and independently to determine the initial themes and subthemes. Subsequently, they met several times to review and reach agreement regarding the themes and subthemes. Third, the researchers reviewed and discussed the themes by reading them again and checking their coherency and consistency with each other and the entire dataset. Finally, the research team met again to define and label the themes and their underlying subthemes and to identify key participant quotations.

## Results

Thematic analysis showed that participants, regardless of their sex, had two main interlocutors for issues concerning romantic relationships and sexuality: close friends and parents. Some participants also reported that, for most romantic and sexual issues, dialogue was sought with their romantic partner. Women identified close female friends as their privileged interlocutors. They actively sought out these friends both if they wanted to share information or tell experiences and if they wanted to compare their experiences with others or solicit advice and obtain a different perspective. In contrast, men tended to rely on both male and female friends. In fact, the possibility of speaking with the latter resulted in their actively seeking these individuals out, reporting that they appreciated a female perspective on matters regarding romantic relationships.

Both men and women shared information with friends about sexuality as a general topic. In their experience, sexuality was a theme that could be freely discussed with friends. Speaking about it made participants feel good because they perceived sexuality as a positive experience and a pleasant aspect of their lives. They specified that they never experienced feelings of anxiety or shame during dialogues with friends on this theme. However, almost all the participants considered their own sexuality and sex life with their partner as part of the private sphere. They reported respecting and paying attention to the privacy of their relationship, rarely telling friends any personal details. Participants said that they kept clear boundaries between social- and relational-level topics, with the latter being protected and kept private, or shared only with their partner.

In contrast, in matters concerning their romantic relationships, parents, along with friends, played an important role. Participants underlined that mothers were sought for advice on this theme. For both men and women, speaking with their mothers appeared to be easier than speaking with their fathers, because the dialogue was perceived as more fluent and less embarrassing. Participants emphasized that while fathers were sought for other matters, they were generally not sought for matters concerning romantic relationships. Indeed, fathers were frequently updated about participants’ romantic experiences by mothers.

Participants reported not engaging in any form of dialogue or advice on sexuality with their parents. In their experience, speaking with parents about this theme was either unnecessary or it made them feel uncomfortable and embarrassed.

Therefore, a variability emerged in the narratives regarding participants’ dialogue concerning romantic issues, whereas a unique and coherent picture characterized the narratives regarding sexuality; only the latter topic was considered entirely private at the relational level.

*“Generally, I don’t get easily embarrassed; if a friend tells me about it, I’m not uneasy. On the other hand, I am discreet about my own experience. I live it as an intimate moment between two people and I don’t like to blurt out to someone else what we have or haven’t done. I’m not judging those who do want to talk about it; but, if there’s no way around it, I stay as vague as possible.”* (Female)

*“It very much depends who you’re talking to; but, I wouldn’t say that I’d get embarrassed. I don’t feel like saying everything... not about certain things… it’s also a matter of rightly respecting my girlfriend’s privacy.”* (Male)

It appears to be a difference in the narratives about sexuality and romantic issues: emerging adults’ narratives about sexuality are all saying that they do not have problems in speaking with their friends about sexuality as a general theme, but they do not share private information about their sexuality with their partner. No variability in the quotations has been found so that thematic analysis was not able to identify specific themes. On the opposite, a larger variability was found among the narratives about romantic issues. The thematic analysis conducted on romantic issues yielded two main themes: “to seek advice” and “to not seek advice.” Each theme comprised several subthemes that corresponded to participants’ perception of how they seek or not advice. During focus group, it is possible that, due to social interactions, an emerging adult changes his/her opinion or adds some details. For these reasons, it is not possible to say that there are two independent categories of people (those who seek and those who do not seek advice). It is better to consider these two main themes as emerging from the social interactions among narratives of the group, in which both are present and characterize the discussion.

In what follows, we provide accurate descriptions of each theme and subtheme by using direct quotations from the focus group participants to better explain emerging adults’ perceptions. An interpreter translated the reported quotations from Italian into English, respecting the original verbal expressions related to the discursive context that they were elicited.

### To Seek Advice

Some quotations revealed that emerging adults, both males and females, share information and seek advice from parents, friends or both. The reasons to do it can be grouped in three different sub-themes.

**“I look for different points of view.”** Some emerging adults’ quotations reported that, in their experience, engaging in a dialogue with close friends and parents was important because it helped them gaining a more complete vision of the romantic experience. Through sharing information and seeking advice, they become aware of different perspectives, thus helping their reflection process:

*“I listen to other people’s opinions mostly to see things from another point of view, because I can’t see clearly into a situation.”* (Female)

*“Sometimes, I realize that I need them to clarify my own ideas. I listen to them because they allow me to see the situation from other points of view or to have a comparison. I do not listen to everybody’s opinion; but, I value the opinions of two or three of my friends and these do have an influence on my perspective.”* (Female)

According to their narratives, through these dialogues, participants could clarify their ideas, especially when the situations they were experiencing were emotionally charged:

*“I think comparison is necessary because in some situations there’s the risk of being too deep into the problem so that one is not lucid about it anymore. I think that one has to choose the people to talk to, otherwise there’s the risk of being an open book. I seek comparison from my mother, as I do like to have a female opinion.”* (Male)

Participants also viewed disclosing issues to parents as an opportunity for self-improvement and personal growth:

*“It is true that, given how I am, I very much need the comparison especially from three friends of mine. To me, it is essential to hear how things are seen from the outside, to have their opinion, because I appreciate them and I also need their help to better myself. This is because I think that, on our own, it’s difficult to meet all the needs of a person or a relationship. On the other hand, more often lately, I need the comparison with my mother and father. […] I talk with friends and with my parents; they’re my family, and I cannot ignore them in what I do.”* (Male)

For these participants, parents seemed to play an important role, because of the guidance they can provide. Parents were often viewed as models, because of their age and the experience they have accumulated over the course of their lives*:*

*“I try to see my parents as role-models… not that I try to imitate them; but, I think they’re two wonderful people… so, I do ask them for advice and opinions.”* (Male)

**“I treasure other people’s words.”** Participants, in some quotations, reported adopting an attitude of thoughtful reflection on close friends and parents’ opinions concerning their romantic relationships. These participants listened to these interlocutors’ advice carefully, and remembered it when necessary. At the same time, they stressed how it is extremely important that they have the final decision on romantic issues. In their experience, the advice could become useful in the future; therefore, they should remember it:

*“In my opinion, it [the advice] comes on time when maybe you’re about to give up; when you’re unsure, it comes to you when you listen to a friend’s advice. It is not true at all that you forget it immediately. In my opinion, advice remains in some sort of stand-by [state] and we pretend we do not know it. When the situation gets tricky, we pull it out and use it!”* (Female)

*“Same for me. Personally, based on my character, I do not immediately forget advice I receive – it sticks. It is not like I find myself doing exactly what they advise me to; but, if I turn to a friend it’s supposedly because I trust her; therefore, I trust the advice she gives too. I’m less categorical when it comes to advice, I listen to them and treasure them; they’re a lot on my mind; they haunt me!”* (Female)

*“I blindly trust my parents’ advice. It’s the one coming from the people who raised me; so, I listen to it. Without taking it as a dogma, I would think about what they told me anyway.”* (Male)

**“I listen and then do it my own way.”** Finally, some participants’ quotations reported that they actively seek dialogue and advice both with friends and parents, but also that it is important that they decide on their own by following their intuition. They recognized that, often, these decisions could bring them to commit mistakes; however, they believed they should experience it independently:

*“I listen to everything, as well, because if I ask for an opinion from certain people it’s because I’m keen on hearing what they think and believe they can point me in the right direction; but, it’s also true that until I myself do not know which way to turn, as long as I have a certain idea I keep going on that route even if everybody tells me they’re doing something wrong.”* (Female)

*“I listen to what my friends say up to a point. I mean, it’s me living my relationship after all; therefore, it’s me doing a bit of soul searching; so, I can take what other people say as an advice, as warnings; but, in the end I will have to try them out. It’s me losing out if I choose the right or wrong thing; so, I listen up to a point.* (Male)

Participants felt a need to listen to advice and opinions when they came from mothers. Often, their mothers would ask questions regarding participants’ romantic relationships, which signaled their desire to be involved in their sons’ and daughters’ lives. It emerged from participants’ narratives that mothers’ solicitation often began during adolescence. Notably, if it was absent during that phase, it did not often begin in adulthood. For participants, without such maternal solicitation in adolescence, it was difficult to self-disclose to mothers during adulthood:

*“In my opinion, parents become important interlocutors when they want to; but, they do it right from the start because now I’m 23-years-old and I would not walk up to my parents and tell them about myself. The fact that she asks questions makes it easier, because I would never talk spontaneously.”* (Female)

### To Not Seek Advice

Some quotations revealed that emerging adults do not share information and do not seek advice from parents and friends. The reasons to do it can be grouped in two different sub-themes.

**“I do not need comparison.”** Some of the emerging adults’ quotations stated that, in this specific phase of life, they did not need to begin a dialogue regarding romantic relationships. They reported not feeling the need or the desire to obtain others’ advice, which made them not seek it out. They emphasized that decisions and issues related to romantic relationships should be dealt with the partner and not by listening to others’ opinions. In their experience, each romantic relationship was unique; therefore, only the partners involved could optimally handle those issues:

*“Based on my personal experience, I’m not asking for opinions anymore. I’ve often noticed that when somebody gives you advice, it’s like they’re projecting what you’re living onto their own lives. They give you advice about what they would do, and everybody just brings up experiences they’ve lived. I don’t find the latter useful, because even if something similar has happened to you and it went in a certain way, it doesn’t mean that things must go in that certain way. Every story is different.”* (Female)

*“You always have to filter out what people tell you about a person you’re in a relationship with, and that filter is the very relationship you have with them, that the others do not know. Other people can only try and explain it from the outside.”* (Male)

Because friends and parents were not perceived as being able to really understand the nature of the romantic relationship and its issues, their advice would not be useful. The sole interlocutor they were interested in was the partner, the only other person who could understand what they were experiencing:

*“After thinking it through on my own a lot, maybe I’ll listen to what they tell me. However, I would still regard it as an external opinion, because it’s never completely consistent with what I think. The opinion that matters most is the one of the person you’re involved with, or in a relationship with. Their opinion is what makes me see if the issue is a real problem or a less important thing. Your friend, your mom, or your dad are external people who may have valuable insights; but, they’re not all-knowing!”* (Female)

Therefore, the need for advice was replaced by a desire to be accepted and supported in a non-judgmental way:

*“I appreciate silent support best. I’m here; if you want we can talk about it; you say what you have to. On its own, advice takes me nowhere. Also, until you face the problem, there’s nothing to do. If you’re involved in a situation, little can be done, because you’d feel like you hadn’t tried hard enough.”* (Female)

Men reported adopting this attitude when they were living a relationship that they defined as stable and serious. Most of them referred being led by the desire to keep reflections and feelings regarding the romantic relationship to themselves in order to protect their partner’s intimacy:

*“If it’s a not-so-serious relationship, I laugh it away with my friends. If the relationship is serious, I do not talk about it with my friends because then everybody knows everything and everybody criticizes you. I don’t think that’s ideal, especially if you care about the girl.”* (Male)

**“I need to choose on my own.”** The need of some participants for personal reflection seemed to be based on the desire to avoid being overly influenced by others’ opinions and ideas. In this case, quotations revealed that participants did not engage in a dialogue with or seek advice from friends and parents, as they thought it was necessary to act autonomously:

*“Even if it means keeping everything to myself and suffer, I prefer to keep everything in because I don’t want that hearing another person’s opinion to influence me or make me understand I was wrong.”* (Male)

In some cases, the need to be autonomous made participants assume a rather extreme position that left no space for compromise. In some cases, previous experiences made them believe that paying too much attention to others’ advice could be risky for their romantic relationships:

*“It’s on them to accept her, if they want to.”* (Male)

*“Back then, I used to give it too much importance. I mean, if I were to go back, it’s possible that I would say goodbye to my friends.”* (Male)

It emerged from participants’ narratives that, sometimes, it is so difficult to listen to others’ opinions and suggestions that they acted in the opposite way, only to demonstrate their decisional autonomy:

*“I think that other people’s opinions go in one ear and come out the other. Plus, it’s not like you do what they recommend, but the total opposite of that.”* (Female).

This attitude emerged in males’ narratives concerning their parents. Opinions that were provided voluntarily provided by the parents, and not solicited by emerging adults, seemed to be a source of negative emotion:

*“Surely, it is the most honest advice; but, it’s the one that gets on my nerves. I cannot point at any one person in the whole world that could give me more heartfelt advice than my mother or father; but, on this topic, I find it very annoying.”* (Male)

When parents tried to make suggestions, or share their opinions, men perceived them as interfering in their private sphere:

*“My parents’ intrusions annoy me a lot. If, on other issues, their advice is more than welcome, I actually may ask for it. On this topic, it annoys me, and if they just have to talk, it goes in one ear and comes out the other.”* (Male)

## Discussion

This study aimed to explore to whom emerging adults talk about their romantic issues, how they do it and whether there are gender differences. Findings revealed that friends are the main interlocutors of emerging adults (DiIorio et al., 1999; Lefkowitz & Espinosa-Hernandez, 2007) and, for someone, the romantic partner him/herself is the main interlocutor, although also parents, in some circumstances, are perceived as important, especially the mothers (Carlson, 2014; DiIorio et al., 1999; Feldman & Rosenthal, 2000). This finding highlights how the network of close relationships in emerging adulthood is greater than one’s family (Amati, Rivellini, & Zaccarin, 2015), and that peers who are living in the same transitional period and probably experiencing the same transitional patterns in romantic relationships function as relevant interlocutors (Collins & Madsen, 2006; Collins & van Dulmen, 2006).

A second important finding was whether and how emerging adults talked about their romantic relationships. The thematic analyses revealed two general positions: seeking advice or not seeking advice. Previous research similarly found that, although emerging adults talk about different topics with their parents and friends, the frequency of talking about romantic topics is lower and increases in the case of difficulties in the relationship (Carlson, 2014; Vallade et al., 2016). The analysis of the participants’ reasons for seeking advice or not, however, provides a more complex picture: some reasons, both within the seeking advice position and the not seeking advice one, are linked to a desire for independence and autonomy, other reasons are linked to the desire to accept the presence of others and to value their contribution. The desire for independence and autonomy is expressed in the subthemes “I listen and then do it my own way” and “I need to choose on my own”, whereas the desire to value others’ contribution is expressed in the subthemes “I do not need comparison”, “I look for different points of view” and “I treasure other people’s words”. Specifically, the desire for independence is expressed in the quotations in which emerging adults underlined the necessity of making their own decisions about romantic relationships. Some of them (“I listen and then do it my own way”) express the idea that close others were important potential interlocutors; however, it is better to make decisions on their own, regardless of whether they were mistaken, in order to strengthen emerging adults’ own capacity for autonomous decision making. In contrast, other quotations (“I need to choose on my own”) express the worry about the possibility of being influenced by close others; therefore, emerging adults actively avoided seeking advice. In other words, their preoccupation concerned the possibility of not being able to express their own opinion or make their own decisions, which made them actively avoid comparison with close others whose advice they saw as intrusive, annoying, and out of place.

On the contrary, quotations in which appears a lack of worry about others intrusiveness (“I do not need comparison”) or, in which others’ contribution is valued because emerging adults are looking for an enriching exchange that could be useful for their autonomous decision-making process (“I look for different points of view” and “I treasure other people’s words”), could revealed that emerging adults feel enough independent to be open to others and their contribution. In these cases, emerging adults are not preoccupied to be excessively influenced by others’ opinion, and they are able to listen to different points of view and still taking autonomous decisions.

Those two different reasons to seek or not advice can be linked to individuals’ autonomy and relatedness needs (Deci & Ryan, 2000), and the developmental process that many emerging adults are facing between individuation and intimacy. Emerging adults who do not want to be influenced by close others, value the importance of their own volition, opinion, and agency in their romantic relationships. They feel the need to stress the importance of their own autonomy, especially regarding their romantic relationships, probably because they are more focused on the transition from the heteronomy of their young life to the autonomy of adult life (Butzel & Ryan, 1997; Kins, Beyers, Soenens, & Vansteenkiste, 2009; Lanz, Tagliabue, Giuliani, Oliveira, & Walper, submitted; Vansteenkiste, Zhou, Lens, & Soenens, 2005).

In contrast, emerging adults who value others’ contribution, seem not to be worried about their independence, it could be that they already feel autonomous, and so they also feel to be able to embrace different points of view: they are probably building an “autonomous-related” self (Kagitcibasi, 2005) whereby talking to others about their romantic relationships was not a threat to their volition, but rather an opportunity for enrichment.

A final interesting aspect emerging from narratives regards how and to what extent emerging adults spoke about sexuality. Participants strongly agreed that sexuality is a topic that could be freely discussed, especially among friends, at least speaking about it in a general sense and not referring to their own relationships or romantic partners. Thus, sexuality is not perceived as a taboo topic, supporting previous findings about the fact that people, during the college years, are more open-minded regarding sexuality (Lefkowitz, 2005). However, all participants maintained that the privacy of their own relationship had to be upheld by others, and they preferred not talking about their own sexual experiences, even with close friends. It is possible that emerging adults did not need to discuss or seek advice on this topic, having already completed the process of sexual socialization (i.e., the phase of acquiring sexual knowledge and values) in adolescence (Bleakley, Hennessy, Fishbein, & Jordan, 2009; Ward, 2003). Adolescents, in fact, are committed to acquire their own perspective and attitudes regarding sexual matters and fulfill this task by communicating about sex mainly with their peers (Bleakley et al., 2009; Prinstein & Dodge, 2008).

Few gender differences were found. Women prefer to talk with female friends, whereas males are interested in talking with both male and female friends. A possible explanation could be that female friends are more able to speak about intimate issues, and this would help males, who previous research found to be more in difficulty in talking about these topics (Korobov & Thorne, 2006; Lefkowitz et al., 2004). Another little specificity was found within the subtheme “I do not need comparison”: males’ quotations revealed that when they are experiencing a stable and serious relationship, they prefer not to talk with friends about the relationship in order to protect their partner’s intimacy.

Despite our interesting findings, there were some limitations or specificities to note. First, the small sample limits the level to which the findings can be generalized. Second, the themes emerged from focus-groups, a research method that allowed us to explore a sensitive topic, however individual interviews or quantitative studies could provide a different picture of the findings. Third, we were interested in the Italian situation because of the lack of studies within this country; therefore, the findings may not be generalizable to other socio-cultural contexts. Moreover, no formal cross-cultural research has been conducted on seeking advice on romantic relationships. Future research should investigate possible cultural differences with regard to these themes. Furthermore, future quantitative studies could investigate whether the reasons for seeking advice are linked with autonomous and relatedness needs in order to confirm or reject our interpretation regarding the reasons expressed in the quotations of the present study.

Overall, our findings indicate that the way that emerging adults seek advice about their romantic and sexual relationships is linked with the transitional phase they are experiencing. In other words, it connects with their developmental changes toward the acquisition of adulthood and psychosocial maturity.
